# Draft Genome Sequences of 10 Bacterial Strains Isolated from Root Nodules of Alnus Trees in New Hampshire

**DOI:** 10.1128/MRA.01440-19

**Published:** 2020-01-09

**Authors:** Ian Davis, Joseph Sevigny, Victoria Kleiner, Kelsey Mercurio, Céline Pesce, Erik Swanson, W. Kelley Thomas, Louis S. Tisa

**Affiliations:** a Department of Molecular, Cellular, and Biomedical Sciences, University of New Hampshire, Durham, New Hampshire, USA; b Hubbard Center for Genome Studies, University of New Hampshire, Durham, New Hampshire, USA; Indiana University, Bloomington

## Abstract

Here, we report the draft genome sequences obtained for 10 bacterial strains isolated from root nodules of Alnus trees. These members of the nodule microbiome were sequenced to determine their potential functional roles in plant health. The selected strains belong to the genera *Rhodococcus*, *Kocuria*, *Rothia*, *Herbaspirillum*, *Streptomyces*, and *Thiopseudomonas*.

## ANNOUNCEMENT

Actinorhizal plants form a symbiotic association with members of the nitrogen-fixing actinobacterial genus *Frankia* that allows these plants to colonize stressed environments (1–6). Besides containing *Frankia* spp., the actinorhizal root nodules contain large numbers of other actinobacteria occupying the same microniche as *Frankia* spp. (7–13). Many non-*Frankia* actinobacteria that have been isolated from actinorhizal root nodules might contribute to nodulation or aid in plant growth and health (8, 9, 14). As expected, this situation is not unique to actinorhizal nodules but is found in all plants that form root nodule structures, including legume nodules (15–17). There is a growing body of evidence suggesting that both wild and cultivated legume nodules are not exclusively inhabited by rhizobia but contain diverse assemblages of nonrhizobial bacteria (15–18).

Root nodule samples were collected from *Alnus* trees found by Adam’s Pond at Jackson’s Laboratory in Durham, NH. The root nodules were surface sterilized with hydrogen peroxide and rinsed several times with sterile distilled water. The last wash of the sterilized nodule was incubated in LB medium to ensure that all epiphytes associated with the plant were removed. The nodule was cut into a fine powder with a sterilized razor, and dilutions were plated onto Czapeck and R2A media containing cycloheximide and nalidixic acid. About 60 isolates were initially obtained, purified, and propagated on either Czapeck or R2A medium (Table 1). These isolates were incubated overnight in their respective isolation medium (Table 1), and genomic DNA (gDNA) was extracted by the cetyltrimethylammonium bromide (CTAB) DNA extraction protocol (19). RNA was removed by RNase treatment. The quality and quantity of the gDNA were verified using a Thermo Scientific NanoDrop instrument. These isolates were initially identified by amplifying and sequencing their 16S rRNA genes. Based on these results, 10 isolates were chosen for whole-genome sequencing analysis to provide insight into their plant-microbe interactions, including potential plant growth-promoting activity.

**TABLE 1:** Genome statistics

Bacterial species	Isolate^*a*^	GenBank accession no.	No. of reads	No. of contigs	Avg coverage (×)	Genome assembly size (bp)	*N*_50_ contig size (kb)	No. of CDSs^*b*^	G+C content (%)	No. of rRNAs	No. of tRNAs
Rothia dentocariosa	1C11	SPNF00000000	4,631,207	97	210.5	2,536,666	1,708.8	2,212	55	10	52
*Streptomyces* sp.	4R-3d	SPNN00000000	8,912,300	93	429.3	8,495,698	213.7	7,601	72	8	65
*Rhodococcus* sp.	1R11	SPNG00000000	5,379,096	38	82.9	5,478,616	346.9	5,102	64	8	58
Kocuria rhizophila	4R-31	SPNK00000000	6,695,169	26	571.5	2,659,245	245.8	2,225	70	9	57
	4R-34	SPNL00000000	2,094,786	122	71.2	2,654,140	39.4	2,211	71	4	46
*Herbaspirillum* sp.	3C11	SPNH00000000	2,844,408	337	186.0	5,552,861	30.9	5,278	60	1	50
	3R-11	SPNI00000000	4,677,477	11	328.3	5,219,991	1,116.6	4,736	59	3	46
	3R11	SPNJ00000000	14,415,312	10	356.0	5,221,161	1,116.6	4,738	60	3	46
	3R-3a1	QUQN00000000	6,064,775	13	170.0	5,221,518	771.5	4,741	59	3	39
*Thiopseudomonas* sp.	4R-3cI	SPNM00000000	6,340,931	405	212.9	2,981,747	15.5	2,995	72	14	65

a C and R represent Czapeck and R2A media, respectively, that were used to culture each isolate.

b CDSs, coding DNA sequences.

Whole-genome sequencing was performed at the Hubbard Center for Genome Studies (University of New Hampshire, Durham, NH) using Illumina technology techniques (20). A paired-end library was constructed using a Nextera DNA library preparation kit (Illumina, San Diego, CA) and sequenced on an Illumina HiSeq 2500 instrument to produce 250-bp paired-end reads. The total numbers of reads for all 10 strains are listed in Table 1. The Illumina sequence data were trimmed using Trimmomatic version 0.36 (21). TruSeq adapters were trimmed with an allowance of two mismatches. Leading and trailing bases below a quality of three were trimmed. The reads were then scanned with a sliding window of 4 bp and trimmed if the average quality dropped below 30. Finally, reads were dropped if the length was less than 36 bp. Trimmed sequencing reads were assembled using SPAdes version 3.13 (22), with the default settings. The assembled genomes were annotated using the NCBI Prokaryotic Genome Annotation Pipeline (PGAP) (23). The assembly metrics and annotation features are given in Table 1. The identities of the strains were determined by whole-genome-based taxonomic analysis via the Type (Strain) Genome Server (TYGS) platform (24) (https://tygs.dsmz.de), including digital DNA:DNA hybridization (dDDH) values (25). The type-based species clustering using a 70% dDDH radius around each of the type strains was used as previously described (26), while subspecies clustering was done using a 79% dDDH threshold, as previously introduced (27). Among the 10 strains, new species of the genera *Streptomyces*, *Rhodococcus*, *Herbaspirillum*, and *Thiopseudomonas* were identified, including one *Herbaspirillum* subspecies. Bioinformatic analysis of these genomes by the use of the antiSMASH 4.0 program (28) revealed the presence of high numbers of secondary metabolic biosynthetic gene clusters. Many of these potential natural products should be involved in plant-microbe interactions and aid in their plant growth-promoting activities.

### Data availability.

The draft genome sequences of these bacterial strains have been deposited in GenBank under the accession numbers listed in Table 1. Both the assembly and raw reads are available at DDBJ/ENA/GenBank under BioProject number PRJNA480027.
